# MS care: integrating advanced therapies and holistic management

**DOI:** 10.3389/fneur.2023.1286122

**Published:** 2024-01-30

**Authors:** Gavin Giovannoni, Helen L. Ford, Klaus Schmierer, Rod Middleton, Andrea M. Stennett, Ian Pomeroy, Leonora Fisniku, Antonio Scalfari, Colin Bannon, Ruth Stross, Sarah Hughes, Adam Williams, Samantha Josephs, Charlie Peel, Agne Straukiene

**Affiliations:** ^1^Centre for Neuroscience, Surgery and Trauma, Faculty of Medicine and Dentistry, The Blizard Institute, Queen Mary University of London, London, United Kingdom; ^2^Clinical Board Medicine (Neuroscience), The Royal London Hospital, Barts Health NHS Trust, London, United Kingdom; ^3^Leeds Teaching Hospitals, University of Leeds, Leeds, United Kingdom; ^4^Disease Registers & Data Research in Health Data Science, Swansea University Medical School, Swansea, United Kingdom; ^5^The Walton Centre NHS Foundation Trust, Liverpool, United Kingdom; ^6^Department of Neurology, University of Liverpool, Liverpool, United Kingdom; ^7^Department of Neurosciences (Addenbrooke’s), Cambridge University Hospitals NHS Foundation Trust, Cambridge, United Kingdom; ^8^Centre of Neuroscience, Department of Medicine, Imperial College London, Charing Cross Hospital, London, United Kingdom; ^9^Retired, Plymouth, United Kingdom; ^10^Neurology Academy, Sheffield, United Kingdom; ^11^Kingston Hospitals NHS Foundation Trust, Surrey, United Kingdom; ^12^Torbay and South Devon NHS Foundation Trust, Torquay, United Kingdom; ^13^Devon Partnership NHS Trust, Paignton, United Kingdom; ^14^Nutritionista, London, United Kingdom; ^15^University of Plymouth, Plymouth, United Kingdom

**Keywords:** multiple sclerosis, brain health, lifestyle, disease progression, outcomes, holistic care, recommendations

## Abstract

Lifestyle and environmental factors are key determinants in disease causality and progression in neurological conditions, including multiple sclerosis (MS). Lack of exercise, poor diet, tobacco smoking, excessive alcohol intake, social determinants of health, concomitant medications, poor sleep and comorbidities can exacerbate MS pathological processes by impacting brain health and depleting neurological reserves, resulting in more rapid disease worsening. In addition to using disease-modifying therapies to alter the disease course, therapeutic strategies in MS should aim to preserve as much neurological reserve as possible by promoting the adoption of a “brain-healthy” and “metabolically-healthy” lifestyle. Here, we recommend self-regulated lifestyle modifications that have the potential to improve brain health, directly impact on disease progression and improve outcomes in people with MS. We emphasise the importance of self-management and adopting a multidisciplinary, collaborative and person-centred approach to care that encompasses the healthcare team, family members and community support groups.

## 1 Introduction

Multiple sclerosis (MS) is an inflammatory demyelinating and degenerative disease of the central nervous system (CNS) affecting the brain and spinal cord (1–3). MS can cause a range of symptoms, including sensory, motor, or visual disturbance, fatigue and cognitive impairment (4). MS can occur at any age, including during childhood and adolescence, with the global average age of MS diagnosis being 32 years (1). MS is a heterogenous disease, with some people having a variable rate of recovery characterised by periods of relapse and remission while others have progressive disease (5–7). Due to the heterogeneity of MS, life for people with the disease and their families and friends can be unpredictable, with negative impacts on their quality of life (QoL) (1). Furthermore, MS is the leading cause of nontraumatic neurological disability in young people, which has major implications for societal costs due to loss of productivity and healthcare costs (1).

Although repair mechanisms and remodelling of the CNS can partially abate the underlying pathological insults in MS, eventually, the compensatory capacity is not temporally aligned with the ongoing inflammatory disease activity (2, 4). Neurological reserve capacity is defined as the ability of the brain to effectively buffer changes associated with normal aging and cope with pathological damage. The baseline reserve is depleted by factors such as aging, neurologic disease progression and health comorbidities, but can be preserved by protective factors such as effective treatments and healthy lifestyles (8). Individual people have varying degrees of reserve at disease onset (9); however, as this reserve is gradually exhausted, the consequences of MS become clinically apparent, with steady increases in physical, mental and cognitive disability. Treatment with disease-modifying therapies (DMTs) can help preserve the neurological reserve and lead to a lower risk of disability (8).

Metabolic health relates to levels of blood glucose, triglycerides, high-density lipoprotein cholesterol, blood pressure, and waist circumference. Lifestyle and environmental factors are key determinants in disease causality and progression in neurological conditions, including MS (10, 11). Lack of exercise, poor diet, smoking, excessive alcohol intake, social determinants of health (SDOH), concomitant medications, poor sleep and comorbidities can exacerbate MS pathological processes by impacting brain health and depleting neurological reserves, resulting in more rapid disease worsening (2, 4). Therefore, in addition to using DMTs to slow down the disease course, therapeutic strategies in MS should aim to preserve as much neurological reserve as possible by promoting the adoption of a “brain-healthy” and “metabolically-healthy” lifestyle (2, 4). Here, we recommend self-regulated lifestyle modifications that have the potential to significantly improve brain health and metabolic health in people with MS and directly or indirectly slow down disease progression and improve outcomes.

## 2 What is brain health?

A recent position paper published by the World Health Organization (WHO) defined brain health as “the state of brain functioning across cognitive, sensory, social–emotional, behavioural and motor domains, allowing a person to realise their full potential over the life course, irrespective of the presence or absence of disorders” (12). Accordingly, brain health is measured as the brain’s ability to grow and build strong neuronal connections, repair neuronal damage and compensate over time in ways that allow people to think, feel, move and interact with the world around them. The WHO identified five major determinants of human brain health optimisation across the life course, namely: physical health, environment, safety and security, lifelong learning and social connections, and access to quality services (12). Brain health can be optimised throughout people’s lifetimes by minimising exposure to risk factors, where possible, and enhancing factors that promote neuroplasticity and recovery from brain injury (12). To optimise brain health in people with MS, it is important to understand how the underlying pathological processes impact brain function, and the resulting sequelae. However, it is also important to identify and optimise modifiable factors that may improve or maintain brain health throughout the disease course. Recommendations for healthcare professionals to maintain brain health in people with MS are shown in Table 1. As the evidence discussed herein shows, it is never too late for people with and without MS to reduce lifestyle risk factors, even for those already experiencing disability.

**Table 1:** Recommendations for healthcare professionals to maintain brain health in people with MS.

Promote prompt diagnosis and early treatment with DMT	Connect with primary care partners (e.g., general practitioners) to ensure prompt referral and early diagnosis and DMT initiation
Discuss infection prevention and treatment	Provide proactive advice to patients with MS on infection prevention and self-management (e.g., good oral hygiene, regular dental check-ups, annual vaccinations, urine self-monitoring for urinary tract infection) and advise people with MS to seek prompt treatment when an infection occurs
Discuss strategies to improve emotional well-being	Encourage people with MS to:
	Retain and increase social connections (e.g., with family, friends, community groups)
	Participate in well-being activities such as meditation, yoga or spending time outdoors
	Consider counselling and NHS talking therapies/CBT
Encourage people with MS to adopt lifestyle changes that preserve cognitive reserve and develop resilience to stress	Encourage people with MS to:
	Remain cognitively active (e.g., reading, continuous learning, creative pursuits, socialising with a variety of people)
	Remain physically active as much as possible (run, walk, swim or any other physical exercise) and encourage pacing through CBT interventions (e.g., behavioural activation)*
	Build their levels of resilience, hope and grit via counselling and involvement with peer support groups and social interactions
	Ensure they get good sleep *
	Eat healthily*
	Maintain good hygiene and balanced fluid intake
	Not smoke*
	Limit their alcohol intake*
	Maintain their emotional well-being*
Ensure regular monitoring of people with MS in MS clinic and via partners (e.g., GP)	Assess general health/comorbid conditions regularly to ensure early identification of conditions that impact vascular health, such as:*
	Hypertension
	Hypercholesterolaemia
	Dyslipidaemia
	Diabetes mellitus
	Hypothyroidism
	High body mass index

*See later section for more specific guidance.

CBT, cognitive behavioural therapy; DMT, disease modifying therapy; GP, general practitioner; NHS, National health System; MS, multiple sclerosis.

### 2.1 Enhance cognitive reserve

Despite MS being considered a disease of young adults, the mean age of the population with MS is increasing, resulting in an increased incidence of MS among the elderly (13). Ageing in people with MS is associated with cognitive impairment, particularly regarding information processing speed and multitasking, as seen in people with dementia due to primary neurodegenerative conditions such as Alzheimer’s disease (14). A similar cognitive profile between young and old people with MS suggests that the cognitive impairment in MS is probably a continuous process starting from the earliest stages of the disease and increasing over its course (14). To slow down brain ageing and maintain neuroplasticity, it is vital that people with MS stay as cognitively active as possible (15). Therefore, people with MS should be encouraged to learn and to connect with others regularly. A short daytime nap has been shown to help with cognitive performance in the workplace (16). High resilience (the ability to achieve, retain or regain a level of physical or emotional health after illness or loss), perseverance and stamina increase healthy ageing and well-being, particularly the ability to maintain healthy lifestyle habits, independence and participation (17, 18).

## 3 Optimising brain health in people with MS

### 3.1 Adopt a multidisciplinary, collaborative and person-centred approach to care that encompasses the healthcare team, family members and community support groups

People with MS have different needs for information, advice and support, and these needs change as MS progresses. Within healthcare services, patients should have a single point of contact who can coordinate access to care from a multidisciplinary team with expertise in MS (19). Care should be tailored to the individual patient and be responsive to their changing needs. To enable and maintain healthy lifestyle behaviours, people with MS may require support from family members and the broader community. Within routine clinical practice, it is crucial to incorporate the ability for healthcare providers to signpost people with MS and their families to local support groups who can assist them with lifestyle behaviours (e.g., smoking cessation support, physical activity programmes, healthy eating advice).

### 3.2 Identify and treat symptoms promptly

#### 3.2.1 Infections

Infections trigger proinflammatory reactions in the brain; preventing infections can positively impact disease progression by minimising inflammatory mechanisms. Mounting evidence from different fields of research supports the pivotal role of the Epstein–Barr virus (EBV) in the development of MS (20). Ongoing research aims to clarify the causative role of EBV in MS; potential mechanisms include the promotion of neuroinflammation via autoimmunity or direct viral infection.

There is increasing evidence that people with MS have a higher risk of overall infections and infections leading to hospitalisation compared with the general population (21, 22). All types of infection are increased, including viral, fungal, pneumonia and influenza, and opportunistic infections (22). Urinary tract infections (UTIs) are the most common infection treated by MS specialists, followed by aspiration pneumonia (22). Poor oral hygiene is reported in some people with MS, and there is evidence that people with MS may be at higher risk of periodontal disease than the general population (23). Proactive infection monitoring, management and self-care education for people with MS, such as being provided with the recently developed home UTI testing kit, can help identify infections early and improve patient well-being, particularly in those with more severe disabilities, who typically have poor outcomes from infection (24, 25). Comprehensive MS bladder management pathways have been developed to increase awareness of the importance of bladder problems in people with MS and how to best manage them.

#### 3.2.2 Bladder and bowel symptoms

**Figure 1:**
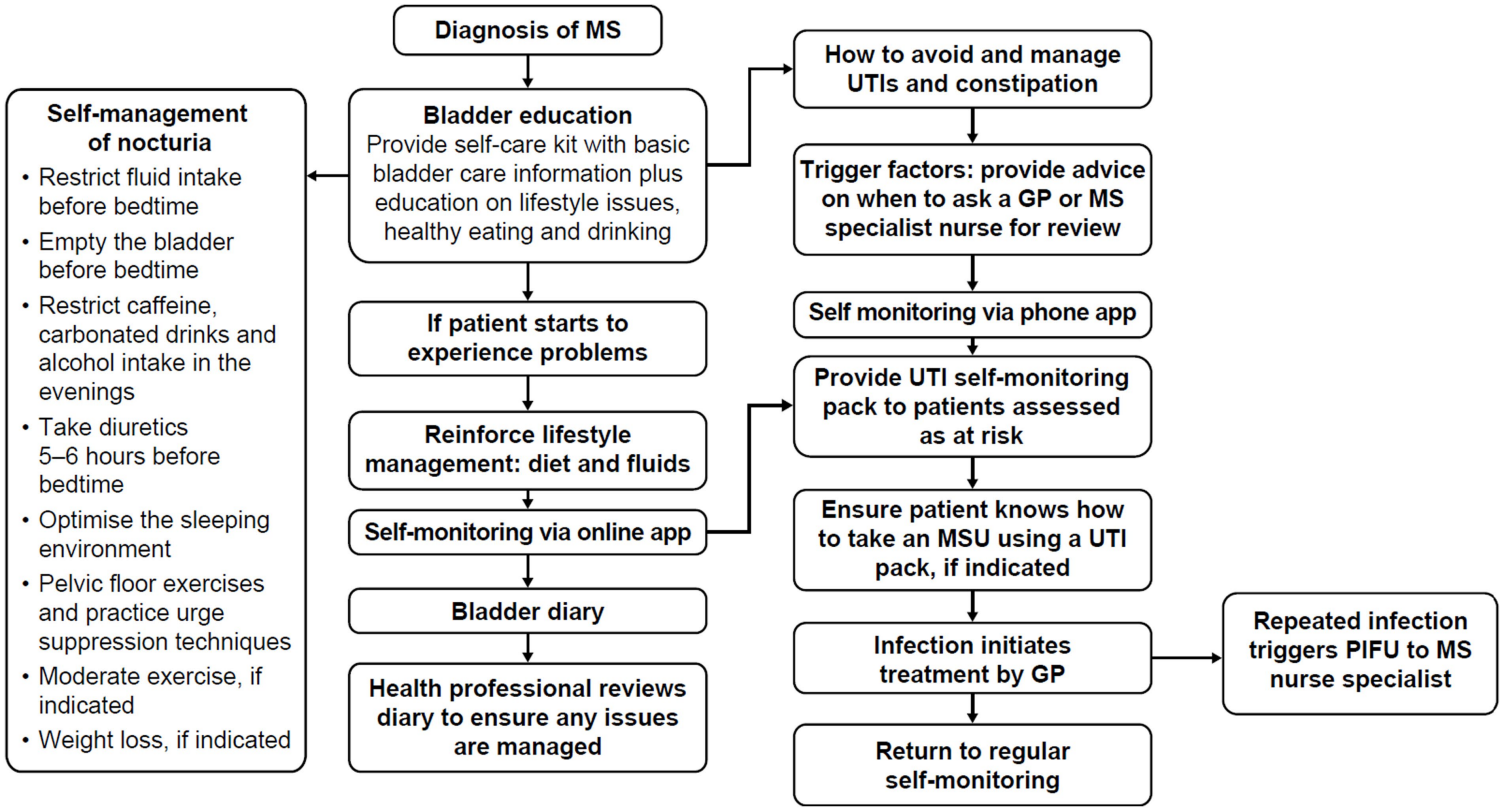
Self-management pathway for bladder dysfunction in people with MS. Adapted from Thomas et al. (25, 46). GP, general practitioner; MS, multiple sclerosis; MSU, mid-stream urine culture; PIFU, patient-initiated follow-up; UTI, urinary tract infection.

Bladder and bowel symptoms have a high impact on QoL (25). Bladder problems contribute to debilitating sleep disturbance (e.g., via nocturia) and are significantly correlated with fatigue, daytime sleepiness and depression. Despite these detrimental impacts, people with MS can be reluctant to seek help, due to a lack of awareness around potential treatments, or the social stigma associated with bladder dysfunction; they may choose to self-manage symptoms alone. Although self-management is recommended to identify UTIs early, other lifestyle modifications such as reduction of bladder irritants like caffeine and alcohol can help to decrease and manage bladder and bowel symptoms, which in turn help to alleviate sleep disruption for some people with MS (Figure 1).

#### 3.2.3 Depression and anxiety

Up to 50% of people with MS are reported to experience depression at some point in their life (26), and the incidence of suicide is higher in people with MS compared with the general population (26, 27). Depressive symptoms in people with MS may be experienced differently by females and males, with females experiencing greater overall symptoms and greater somatic symptoms compared with males (28). Social isolation may increase the risk of depression and anxiety in people with MS; loneliness is reported by 60% of people living with MS (29, 30).

Many factors play a role in developing depression in people with MS, including a link to increased inflammatory activation of the immune system affecting both the periphery and the CNS. Chronic inflammation caused by stress has a causative association with chronic diseases, including depression (31). In addition, a role for immune activation in depression is supported by response to treatments targeting immune pathways, which are effective in treating depression and fatigue in some cases (17).

Depression can be very disabling; it affects QoL, productivity and brain health (32). However, low mood is not necessarily related to depression. Depression and physical symptoms of MS can be quite similar, e.g., brain fog, lower motivation for certain activities, reduced energy levels or withdrawal. Moreover, people with depression do not always realise that they have depression – in fact, other family members may notice signs first (33). It is important to identify early signs of depression and obtain a clinical diagnosis as soon as possible, as depression can lead to increased symptoms and decreased QoL in people with MS (32). There is a vicious cycle between (1) depression worsening MS symptoms (through inactivity and negative automatic thoughts), and (2) the physical impact of MS symptoms worsening depression. This can be treated with psychological therapies such as cognitive behavioural therapy (CBT), acceptance commitment therapy (ACT) and counselling.

Anxiety is reported to occur in up to 45% of people with MS (34). Females and people with a relapsing–remitting MS (RRMS) disease course and a worse degree of functional disability show higher rates of anxiety symptoms than their counterparts (34). As with depression, psychological therapies such as CBT, ACT and counselling are treatment options.

#### 3.2.4 Cardiovascular symptoms

Vascular damage in the brain may contribute to neurodegeneration and is associated with lower cognitive function in both people with MS and in the general population (35). A recent UK study reported that at the time of MS diagnosis, people with MS have an increased prevalence of vascular risk factors, including hypertension and diabetes, compared with matched controls (36). It is therefore important to prevent, or identify and control, conditions that increase the risk of cardiovascular disease, including diabetes mellitus, hypothyroidism, hypercholesterolaemia, dyslipidaemia, hypertension, sedentary lifestyle and obesity. The impact of comorbid conditions in people with MS is discussed in more detail later in this review.

#### 3.2.5 Sleep disorders

Sleep disorders are more prevalent in people with MS than in the general population and include sleep-related breathing disorders, restless legs syndrome, periodic limb movement disorders, secondary narcolepsy, rapid eye movement sleep behaviour disorder and propriospinal myoclonus (37). Magnetic resonance imaging (MRI) has shown a link between sleep disorders and lesions in specific CNS regions (37). Management of sleep disorders can be complex in people with MS, and multidisciplinary care, with referral to sleep medicine specialists and other specialities (e.g., urologists), may be required.

Changes in sleep quality over time impact how people with MS experience a whole host of symptoms, and there is evidence that poor sleep quality may be a trigger for MS relapse and can negatively influence MS outcomes (38). There is a complex relationship between MS symptoms and sleep, with some symptoms exacerbating others, leading to a vicious cycle of sleep disruption, whilst some known MS symptoms may themselves be caused by a lack of sleep (39) Because pain sensitivity may be heightened by sleep loss (40), researchers recommend the management of sleep and anxiety in people with MS to decrease their pain levels (41).

**Figure 2:**
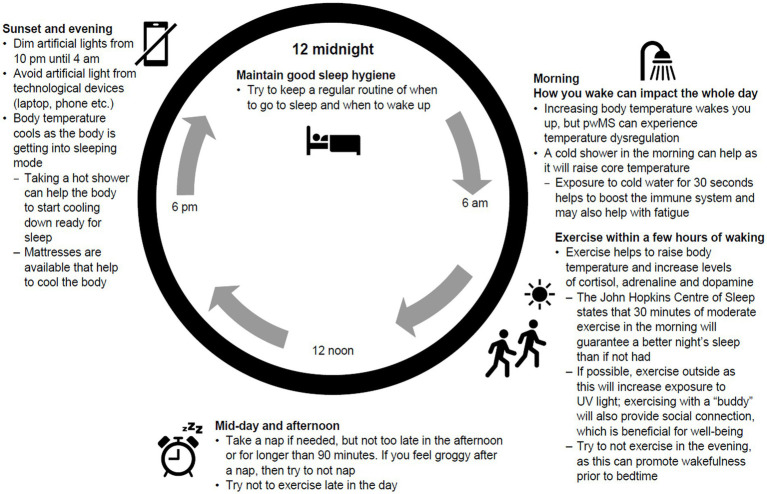
Guidance on good sleep hygiene and sleep architecture. UV, ultraviolet.

Education around good sleep hygiene and the impact of lifestyle choices on sleep quality need to be available to people with MS, with CBT offered for those with chronic insomnia (42). Self-management of sleep disruption should be encouraged by helping people with MS understand the circadian rhythm and how body temperature, light exposure and nutrition can impact sleep architecture (Figure 2). Tailoring sleep hygiene advice to the individual is important for those with MS, considering symptoms, medication and side effects, and ensuring information on physical activity, natural light and nutrition are personalised (43–46).

Medications, including DMTs and symptomatic medications (e.g., baclofen, gabapentin, oxybutynin), can affect sleep in people with MS either by causing insomnia or hypersomnia; the underlying mechanisms vary [Table 2; (47–49)].

**Table 2:** Medications commonly used in the treatment of MS with the potential to disrupt sleep and proposed adaptations to avoid or decrease sleep disruption.

Medication	Indication in MS	Effects on sleep	PSG data	Proposed MoA	Adaptation
DMTs					
Interferon-beta	First-line DMT	Fatigue, hypersomnolence, insomnia	Reduction of sleep efficiency	Reduces antigen presentation and T-cell proliferation; alters cytokine expression	Switch from evening to morning injection
Glatiramer acetate	First-line DMT	More frequent awakenings and daytime somnolence; increased anxiety and irritability	Not available	Partly due to increase in anti-inflammatory cytokines (IL-4 and IL-10) through activation of the Th-2 cell pathway of immunity	
Natalizumab	Second-line DMT	Improvement in fatigue, daytime sleepiness and depression	Not available	Monoclonal antibody against the α-4 subunit of α-4 β-1 integrin that inhibits the ability of activated T cells to migrate across the blood–brain barrier into the CNS	
Symptomatic medications					
Methylprednisolone	Acute relapse	Insomnia	Decreased REM sleep	Decreased cytokine cascade, activation of B and T cells and ability of immune cells to penetrate the CNS	Lifestyle changes, CBT, pharmacological intervention (if needed)
Modafinil	Fatigue	Insomnia	Reduced sleep latency	Unknown	
Methylphenidate	Fatigue	Insomnia	REM sleep suppression	Increased catecholamine release and reuptake inhibition	
Amantadine	Fatigue	Insomnia	Not available	Presynaptic dopamine release	
Pemoline	Fatigue		Not available	CNS stimulant	
4-Aminopyridine	Fatigue	Insomnia	Not available	Block of K channels in neurons	
Baclofen	Spasticity	Sedation	Total sleep time increased and reduced wake after sleep onset	GABA-B receptor agonist	
Clonazepam	Spasticity, anxiety	Somnolence	Increased total sleep time, reduced sleep latency and wake after sleep onset, increased spindle activity and reduced REM sleep	GABA-A receptor agonist	
Tizanidine	Spasticity	Daytime drowsiness	Improvement in sleep induction and maintenance	Central α-2 adrenoreceptor agonist	
SSRI	Depression, anxiety	Insomnia or sedation	Decreased total sleep time, increased stage 1 sleep, decreased REM sleep, increased sleep latency, “Prozac eyes” and periodic limb movements	Inhibition of serotonin reuptake	
Gabapentin	Pain seizures	Sleepiness	Decreased sleep stage 1, increased sleep stage 3, reduced periodic limb movements, increased REM sleep	May promote formation of GABA in the CNS	
Oxybutynin	Urinary frequency	Sedation	Decreased REM sleep and increased REM sleep latency	Anticholinergic agent	
Cannabis-based medicinal extracts	Spasticity, bladder dysfunction, central neuropathic pain	Improved sleeping difficulty and sleep quality, diminished awakenings and sleepiness	Not available	Inhibition of smooth muscle contraction, interaction with the cholinergic receptor system and/or synergism with anticholinergic medication, analgesic properties	

Adapted from Brass et al. (47) and Lanza et al. (48). CBT, cognitive behavioural therapy; CNS, central nervous system; DMT, disease-modifying therapy; GABA, gamma-amino-butyric acid; IL, interleukin; K, potassium; MoA, mechanism of action; MS, multiple sclerosis; PSG, polysomnography; REM, rapid eye movement; SSRI, selective serotonin reuptake inhibitor.

#### 3.2.6 Comorbidities

Certain comorbidities are more prevalent in people with MS than in the general population, including cerebrovascular, cardiovascular, neurological and musculoskeletal diseases, psychiatric disorders and thyroid disease (50–52). Comorbidities influence the course of MS, have medical and socioeconomic consequences (50) and also have impact on the treatment response and on switching between treatments, as they are associated with lower persistence on DMTs (51). To mitigate the challenges of comorbidities, it is important to monitor their occurrence in the MS population and to consider the medical and social consequences of these different conditions.

The most common comorbidities in people with MS admitted as inpatients in England during the financial year 2020/2021 (*n* = 31,275) were hypertension (24.8% of admissions), abnormality of gait and mobility (likely related to musculoskeletal conditions; 17.1%), disorders of the urinary system (other than MS-related; 15.3%), depressive episode (13.9%), type 2 diabetes (11.7%), smoking (10.9%), asthma (10.9%), other anxiety disorders (9.0%), obesity (8.9%) and chronic ischaemic heart disease (7.5%) (53).

The relationship between comorbidities and MS is complex: they may be due to a direct causal relationship, common risk factors for disease or a secondary association (50). For example, thyroid disorders appear to affect people with MS more than the general population. However, thyroid dysfunction is a known side effect of some DMTs, particularly alemtuzumab and, to a lesser extent, interferon-beta. It is, therefore, important to assess the true causal association (50).

Issues related to comorbidities should be part of patient counselling, especially regarding lifestyle factors, as preventing or effectively treating comorbidities can alleviate the burden of MS (Table 3) (50). Furthermore, differential diagnosis between MS symptoms and symptoms caused by other, undiagnosed conditions is essential to ensure effective management and avoid increased disease burden. Without specific screening programmes in general practice, monitoring of weight, blood pressure and relevant blood tests should take part in secondary care.

**Table 3:** Recommendations to identify and manage comorbid conditions in people with MS.

Regularly assess comorbid conditions in people with MS and take proactive steps to improve conditions where possible	Issues related to comorbidities should be part of counselling, especially regarding lifestyle factors, as preventing or effectively treating comorbidities can alleviate the burden of MS	Promptly identify comorbidities by asking people with MS about their general health at all consultations	Refer people with MS to appropriate specialties/pathways or members of the multidisciplinary team
Provide practical guidance on how to live well with MS and comorbid conditions	Recommend healthy lifestyle measures to prevent or alleviate comorbidities associated with obesity, smoking, physical inactivity, poor diet, and menopause	Signpost to services that can assist with each comorbid condition	Highlight the importance of keeping up to date with nationally recommended health screening

MS, multiple sclerosis.

#### 3.2.7 Life stage—Menopause and multiple sclerosis

Since MS is typically diagnosed in patients aged 20 to 40 years old, most women living with MS will undergo menopause after MS diagnosis (54, 55). It has been reported that in women with MS, menopause is associated with a reduced ARR, but an increase in disease-related disability (56, 57). However, the effect of menopause on MS management and progression is not fully understood, as most randomised controlled trials are not powered to evaluate DMTs in menopausal populations (58, 59).

Menopause, which typically occurs in women between the ages of 45 and 55 years, is a normal physiological transition caused by the loss of ovarian follicular function and a decline in circulating oestrogen levels (60, 61). Perimenopause, which often lasts for several years, refers to the period from when these symptoms are first observed and ends 1 year after the final menstrual period. Some women experience premature menopause before they reach 40 years, which may be due to certain chromosomal abnormalities, autoimmune disorders, or other unknown causes (60). Symptoms of the perimenopause often include hot flushes, night sweats, worsening mood, depression and/or anxiety, worsening fatigue, worsening bladder control, and difficulty in sleeping/insomnia (60).

MS is more than twice as common in females than in males (62). Following diagnosis, MS will impact women through all stages of their life, including puberty, family planning, pregnancy and childbearing, breast feeding, infertility treatment, menopause and cancer prevention (e.g., vaccination against papillomavirus, smear tests to detect cervical cancer) (63, 64). Several immunologic changes have been reported in post-menopausal women, including decreased CD4 T lymphocytes, B lymphocytes and natural killer cells cytotoxic activity, and increased proinflammatory responses, which all have effects on infection and autoimmunity. These immunologic changes overlap with age-related changes and are driven by oestrogen deprivation.

Some studies have shown a shift in MS onset to an older age, with a higher frequency of late-onset forms in women; the possible effect of menopause on the susceptibility to MS should be considered in these patients (59). This effect may be attributable to the postmenopausal proinflammatory state and deprivation of the neuroprotective effects of oestrogen and progestins, which can reduce the resilience of the CNS and thereby facilitate the onset of MS when other predisposing factors are present (58, 59).

The hormonal changes during perimenopause can affect physical, emotional, mental and social well-being. The symptoms of MS and perimenopause can frequently overlap, including fatigue and disturbances in cognition, mood, sleep, bladder and sexual function (55). This can create challenges in ascertaining the likely cause of symptoms to be treated. Once perimenopause or menopause is diagnosed, an explanation of the stages of menopause should be provided, along with the benefits and risks of treatments for menopausal symptoms. Some strategies have been suggested for managing menopause and MS-related symptoms simultaneously (Table 4) (65). It is important to use a holistic approach to healthcare that addresses both conditions.

**Table 4:** Recommendations to identify and manage the menopause in women with MS.

Provide guidance for women with MS who are menopausal	Offer hormone replacement therapy to women with MS who are experiencing menopause
	Refer women to a healthcare professional with expertise in menopause if treatments do not improve their menopausal symptoms or they have ongoing troublesome side effects
Suggestions for managing symptoms of the menopause and MS-related symptoms simultaneously	Hot flushes: Wear layers of light clothing which can be removed easily if necessary; use a handheld fan or cooling spray; avoid potential triggers such as caffeine, alcohol, smoking and spicy foods; exercise regularly; and have cold drinks.
	Night sweats: Sleep in a cool and well-ventilated room; Keep a cold drink by the bedside; take a cool or lukewarm shower; put a towel on your bed if necessary; wear light clothing to bed; try a cooling mattress topper or pillow
	Low mood: Make sure you are getting enough sleep; exercise regularly; participate in relaxing activities such as yoga or meditation; talk about your feelings; do something you enjoy every day.
	Reduced sex drive: Talk to your partner about how you are feeling; take it slow and make more time for foreplay; try to relax by doing some breathing exercises or practising mindfulness.

MS, multiple sclerosis.

Hormone Replacement Therapy (HRT) can be used to supplement hormones that are lost during the menopausal transition, and conventionally contains both oestrogen and progesterone components (66). Hormone replacement therapy can be offered to women with MS experiencing menopausal symptoms (67). A survey focusing on the changes in severity of MS symptoms with the menopause and use of HRT showed that although MS symptoms worsened with the menopause, 75% of respondents who had tried HRT reported an improvement in symptoms (68). HRT has also been associated with better physical quality of life in postmenopausal women with MS (69). In addition, a phase 2 study (n = 164) found that women who took oestriol with glatiramer acetate reduced the number of relapses compared with women who took only glatiramer acetate, although this reduction did not meet statistical significance (70). Despite these results, HRT has not shown consistent benefits in patients with MS, and patients should consult their healthcare practitioners for personalized advice on HRT use (71).

It is important to acknowledge menopause as a significant aspect of MS care and to manage related symptoms. Optimizing sleep quality, mood and hot flush quantity/interference could substantially improve mental health in menopausal women with MS (61). As such, healthcare providers should be encouraged to consider menopause when tailoring treatment plans for women with MS.

### 3.3 Early treatment with DMT and lifestyle interventions

Accumulation of disability in MS may occur as relapse-associated worsening or steady progression independent of relapse activity (72). Studies have consistently shown a correlation between the number of relapses in the first years of MS and disability accumulation in the long term (73). Furthermore, a shorter time interval between the first two relapses predicts faster disease progression (4, 73). In line with these epidemiological observations, large numbers of MRI inflammatory lesions at disease onset and accumulation over the first 5 years were shown to be correlated with poor prognosis (74). Overall, these data support the notion that early focal inflammatory activity contributes to faster brain degeneration in the long term (75). Indeed, evidence shows that implementation of early aggressive treatment with DMTs is correlated with significant improvements in clinical metrics of disease, including relapse rate, disability scores and MRI activity; however, its impact on a patients’ overall QoL is limited, suggesting MS may still subtly progress over time despite effective control of inflammatory activity (2, 76). Consequently, effective DMT and a “brain-healthy” lifestyle should be initiated as soon as the disease is diagnosed to protect the neurological reserve and to maximise lifelong brain health (4, 77). This has recently been further underpinned by the adoption, for the first time, of MS DMT to the World Health Organization’s Essential Medicines List (78).

#### 3.3.1 Movement and physical activity

Movement involving a range of physical activities, including exercise, is recommended as part of the symptomatic management of MS. The beneficial effects of movement and physical activities are well documented and include improved muscle strength (79), increased mobility (80), improved QoL (81), reduced fatigue (82) and reduced depression (83). Therapeutic modalities vary from aerobic exercises, aquatic exercise, balance and resistance training, yoga, tai chi and Pilates to virtual reality and video games (83, 84). Aquatic exercise may lead to increased blood flow to the brain and increased levels of the anti-inflammatory brain-derived neurotrophic factor (84). Furthermore, aquatic exercise may help to improve mobility and maintain body temperature, which may help to counter any temporary exacerbation of MS symptoms due to Uhthoff’s phenomenon (a worsening of neurological function for <24 h in response to increases in body temperature) (85). To optimise the benefits associated with movement, activities should be regular, include the FITT principles (Frequency, Intensity, Time and Type) and be meaningful to the person with MS to facilitate compliance (Table 5) (86).

**Table 5:** Recommendations to increase or maintain exercise and physical activity in people with MS.

Provide guidance on how to maintain an active lifestyle and engage in physical activity at all stages of MS – work with partners (e.g., physiotherapy, healthcare providers, community and charity-based organisations) where needed	Perform a tailored assessment adapted to individual needs
	Review and adapt exercise and physical activity programmes as required (e.g., owing to changes in disability levels)
	Encourage and support people with MS to engage in regular physical activity, whatever their level of ability
	Engage with the person with MS to find the activities that they enjoy and are meaningful to them. This strategy might help to motivate and sustain their activity levels over time
	Encourage people with MS to start small and build to the recommended physical activity levels based on their disease course

MS, multiple sclerosis.

**Figure 3:**
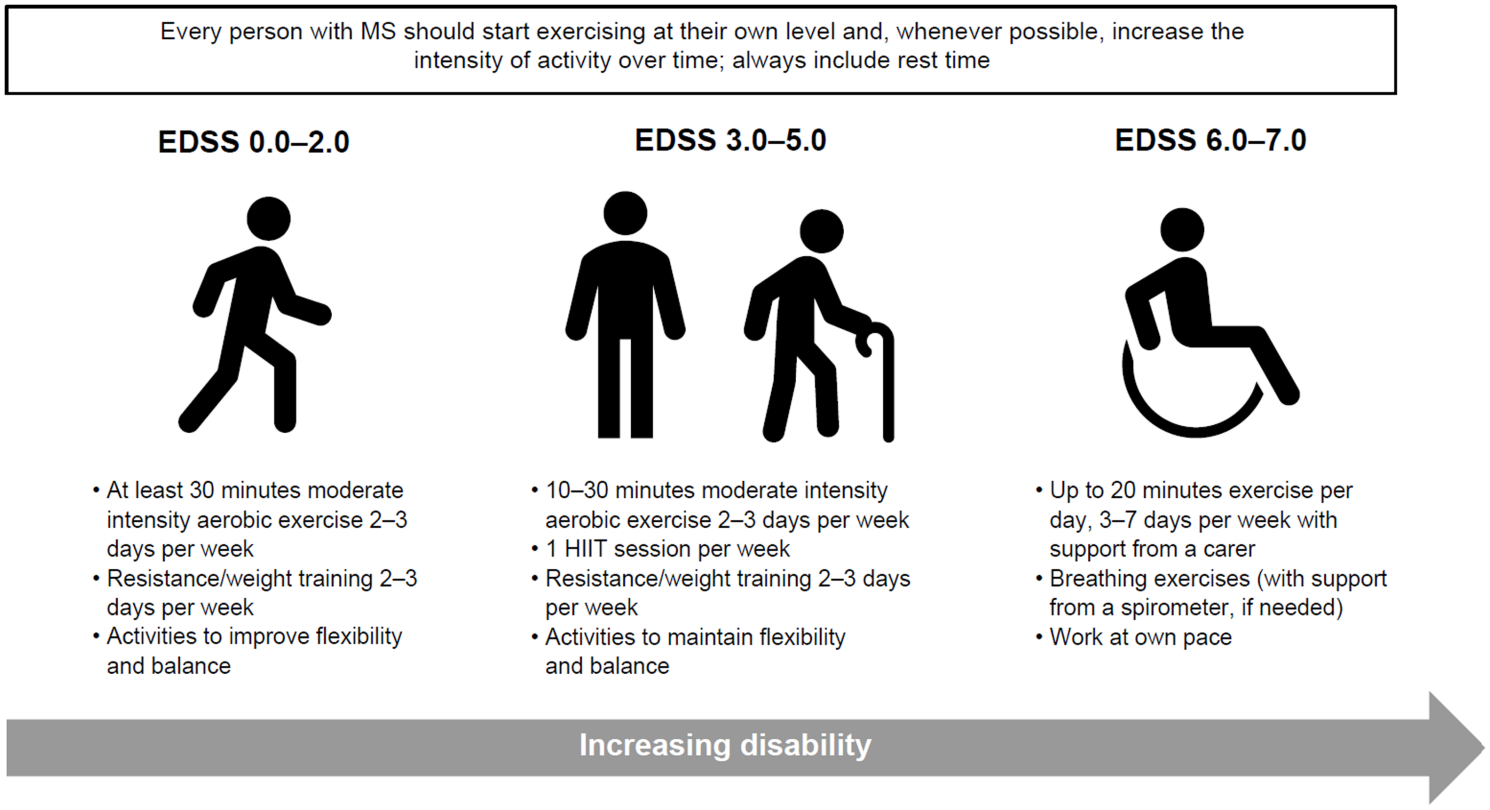
Guidance on minimal amount of physical activity for people with MS to improve or maintain function, reduce disease activity and prevent disability. Adapted from Kalb et al. (87). EDSS, Expanded Disability Status Scale; HIIT, high intensity interval training; MS, multiple sclerosis.

Clinical studies investigating the impact of movement and physical activity on people with MS tend to focus on more mobile individuals, with fewer studies on those with limited mobility; however, physical activity is important, and if possible, should be promoted for all people with MS, irrespective of their disability levels. In previously inactive people, it is important to start exercise as early as possible after MS diagnosis to reduce long-term disability. Therefore, people with MS should be encouraged and supported to participate in physical activity throughout their MS journey, and to incorporate suitable exercises or stretches into their daily life (Figure 3). Similarly, healthcare providers and stakeholders can create opportunities to promote and embed physical activity within MS care to prevent the negative consequences of inactivity and optimise brain health.

#### 3.3.2 Diet and nutrition

The composition, structure and function of the brain are dependent on the availability of appropriate nutrients, in particular lipids, amino acids, vitamins and minerals. It is, therefore, logical that food intake and quality have an impact on brain function. In addition, endogenous gut hormones, neuropeptides, neurotransmitters and the gut microbiota are affected directly by the composition of the diet (88). The emergence of the new research field of nutritional psychiatry offers promise in identifying which dietary components are most important for mental health and for whom, under which circumstances and at which specific dosages these nutritional interventions have preventative and therapeutic efficacy.

Three aspects of MS pathophysiology can be impacted by diet and have the potential to affect disease outcomes, which are: modification of the inflammatory state, protection against neurodegeneration and promotion of injury repair (89).

A wide range of nutrient and dietary approaches have been studied in people with MS, but definitive recommendations are limited owing to the limited quality, duration and scope of many trials, and the inherent challenges of conducting and interpreting nutrition research (89–94). Conclusive evidence supporting the notion that dietary changes can impact disability levels are lacking, but meta-analyses have shown improvements in QoL and fatigue (89, 91, 95). Limited evidence suggests that calorie restriction may help to achieve weight loss in people with MS and may improve emotional health (96). In addition, as people with MS are at greater risk of cardiovascular disease (97, 98), it would be prudent for doctors to stress the use of a diet which controls sources of saturated fat and fried food.

Nutritional psychiatry studies have reported that high fruit and vegetable intake increases happiness, mental health and well-being (88). Diets high in fruit, vegetables, fish and whole grains and the Mediterranean diet have also been linked to reduced risk of depression and reduced risk of dementia, and intake of antioxidant polyphenols in the elderly has been associated with improved cognitive abilities (99, 100). High-fibre diets and a Mediterranean diet promote a diverse gut microbiota and are associated with a reduced likelihood of depression (88). Meanwhile, a processed and ultra-processed Western-style diet is associated with an increased risk of mood disorders, cognitive impairments (particularly memory impairments) and increased anxiety-like behaviour (101). Fibre (<30 g/day), red meat (>70 g/day) and saturated fat (>20 g/day) consumption in women with MS was associated with worse QoL outcomes compared with a control population who were not diagnosed with MS (102). The recommendations to improve diet and nutrition are shown in (Table 6). Although the above-mentioned diets have shown some benefits for people with MS, the results are not yet conclusive and further study is required. Specific, high-quality diets created for people with MS may be a valuable resource for patients, providing a framework for patients who want more information on healthy eating as well as a support network (103, 104).

**Table 6:** Recommendations to improve diet and nutrition in people with MS.

Discuss different diets that have shown potential benefits on health outcomes	Provide information on the Mediterranean diet, low glycaemic load/ketogenic diet and intermittent fasting
	Ketogenic diets should not be followed long-term due to detrimental effects on the gut microbe, possible vitamin deficiencies, kidney stones, etc
	Encourage people with MS to eat a high-quality diet:
	Eat a wide variety of organic, seasonal, fresh, local, and plant-based foods
	Regularly include oily fish (e.g., salmon, sardines, mackerel and trout), nuts (e.g., almonds, walnuts, Brazil nuts, cashew nuts and hazelnuts), seeds (e.g., pumpkin, flax, sesame and sunflower seeds), legumes (e.g., lentils, peas, chickpeas, peanuts), and healthy oils (e.g., olive and flax oil)
	Switch from refined to whole-grain foods (e.g., from white to whole-grain rice, bread and pasta)
	Eat plenty of fibre, including fruit and vegetables (ideally, fresh or frozen fruit and vegetables)
Provide details of foods to avoid	Avoid: excess salt, processed and ultra-processed foods and deep fried, takeaway and fast “junk” foods
Provide practical guidance to help adopt a healthy diet	Adopt regular mealtimes and reduce snacking
	Stay hydrated; drink 1.5 L water, herbal tea or infusions daily
	Reduce sugar intake by reducing or avoiding soft drinks, confectionary and added sugar in hot drinks
	Reduce the burden of cooking by bulk cooking on good days and then freezing portions that can be eaten on days when MS symptoms are more disabling
	This can also be very cost effective
	Invest in gadgets that can help with food preparation and mechanisms to alleviate fatigue in the kitchen (e.g., electric tin opener, food processer, perching stool)
	Use a slow cooker so that meals can be prepared early in the day to avoid end-of-day fatigue
	Buy ready-prepared frozen vegetables and fruit, which are nutrient-rich but also labour-saving and cost effective
	Avoid large meals, particularly those rich in carbohydrates

MS, multiple sclerosis.

#### 3.3.3 Tobacco smoking and alcohol

Tobacco smoking is a risk factor both for developing MS and for more rapid disease progression and higher levels of disability (105–108). The relative risk for MS development is approximately 1.5 for tobacco smokers compared with non-smokers (105, 108). Several studies have reported associations between current smoking and MS progression, worsening of clinical symptoms and neurological disability, higher rates of brain atrophy and decreased time to conversion from RRMS to secondary progressive MS (106, 107). In addition to the direct impact of smoking on MS pathogenesis, there is an association between tobacco smoking and depression and anxiety in people with MS (107). Although vaping is less harmful than smoking cigarettes and is a useful tool for smoking cessation, the long-term risks are unknown (109).

The impact of tobacco smoking on long-term grey matter atrophy and clinical disability was studied in people with RRMS (106). After 10 years, tobacco smoking was associated with lower total white matter volume and higher logT2 lesion volume, lower deep grey matter volume and more severe walking impairment. Data from the UK MS Registry showed that over 8 years, the rate of motor deterioration was accelerated in tobacco smokers vs. non-smokers (110). In addition, over the same period, the rate of motor deterioration reduced to the rate observed in non-smokers in those who gave up tobacco smoking. This study shows that it is never too late for people with MS to benefit from giving up tobacco smoking. A good example of a designated programme to help people quit smoking or reduce/stop alcohol intake is the Better Health programme run by the NHS in England and Wales, which includes access to apps designed to assist with smoking cessation and reducing alcohol intake (111).

The relationship between alcohol and MS risk is heterogeneous between studies, but there is a lack of evidence to support a protective effect of alcohol on MS risk in the UK population (112). However, in general, alcohol in excess has detrimental effects on health, and this also applies to people with MS (4). It is, therefore, prudent to use a screening tool to assess whether drinking behaviour in people with MS is pathological (113, 114).

There is a need for large, prospective, longitudinal trials to investigate how alcohol intake impacts MS disease incidence and progression and how alcohol drinking behaviour changes with disease activity over time. Recommendations to reduce smoking and alcohol intake in people with MS are shown in Table 7.

**Table 7:** Recommendations to decrease smoking and alcohol intake in people with MS.

Ascertain smoking habits and alcohol intake in people with MS	Use a suitable screening tool, such as the Alcohol, Smoking and Substance Involvement Screening Tool – Lite (ASSIST-LITE) to identify risky alcohol and drug use and tobacco smoking
	Regularly perform a suitable alcohol screening test to assess drinking behaviour in people with MS
Discuss poor outcomes associated with smoking and alcohol	Advise people with MS to stop smoking as soon as possible – it is never too late to quit smoking, even for people with MS
	If a person with MS is unwilling to stop smoking or finding smoking cessation difficult, advise them to reduce their amount of smoking as much as possible
	Abstain from drinking alcohol, if possible, or maintain intake in line with recommended weekly allowance for the general population
Provide practical guidance to help people with MS reduce or stop smoking/alcohol	Emphasise the significant cost–benefit of giving up or reducing smoking/alcohol intake
	Suggest that people with MS avoid the environment in which they usually smoke or drink while maintaining social contact (e.g., meet a friend for a walk or for a coffee rather than in a bar or a pub, meet in the daytime rather than in the evening)
	Recommend that people with MS sign up to a designated programme to help them quit smoking or reduce/stop alcohol intake
	For example, the Better Health programme run by the NHS in England and Wales, which includes access to apps designed to assist with smoking cessation and reducing alcohol intake

MS, multiple sclerosis; NHS, National health service.

#### 3.3.4 Emotional well-being

There are multiple evidence-based methods used to alleviate depression and anxiety in people with MS, including CBT, ACT, exposure therapy, mindfulness, virtual reality and music therapy (115–118) (Table 8). Enhanced physical activity and self-efficacy are also important means by which healthcare professionals can help people with MS to reduce their levels of anxiety (119). However, there is no definitive solution, and different approaches will likely suit different people. A person-centred approach to care is key.

**Table 8:** Recommendations to improve emotional well-being in people with MS.

Discuss the prevalence of depression and anxiety in people with MS and their consequences on outcomes	Challenge the person’s preconceived ideas of MS and what it means to them and their lifestyle, life goals, etc.
	Treat every person with MS as an individual and find the most effective approach for that person (e.g., CBT, ACT, mindfulness)
	Promote early access to psychology services for people with MS and identify local well-being programmes, including local mental health services, local and national charities and community support groups
Provide practical guidance to help people with MS reduce the likelihood of depression and anxiety	Promote self-management – arm people with MS with techniques that they can implement in their everyday life
	Help the individual adapt their lifestyle rather than change it completely – this will help them to feel in control of their life following their MS diagnosis or disease progression
	Provide details of MS charities and organisations that run programmes on mental well-being (e.g., MS Society, Shift MS, MS Trust, ACT MySelf)
	Provide avenues into communities of people living with MS, so that people with MS can share their experiences and provide support to each other, these can be online or in person (e.g., Overcoming MS Circles, Shift MS forums, Health Living Clinics)
	Encourage social connectivity, physical exercise, a healthy diet and no smoking

ACT, acceptance commitment therapy; CBT, cognitive behaviour therapy; MS, multiple sclerosis.

### 3.4 Assess social determinants of health

Although many of the lifestyle or behaviour changes mentioned above in relation to brain health are important considerations, many social determinants of health (SDOH) also affect people with MS and these often cannot be changed. SDOH are the conditions in which people are born, grow, live, work and age. These circumstances are the non-medical factors that influence health outcomes. It has been suggested that SDOH account for 30 to 55% of health outcomes (120). According to the World Bank, as of 2022, over one-third of the global population (3.1 in 8.0 billion) resides in low-to-middle-income countries (121, 122) where healthcare inequities and lack of access to quality care are key challenges. Access to healthcare is not only a feature of low-to-middle income countries, with access in high income countries being impacted by different insurance models or the structure of the healthcare systems (123–125).

SDOH may be specific to the individual: sex, gender, race, ethnicity, education, employment, socioeconomic status, safety (e.g., presence of domestic violence and abuse or local crime rate) or to the local social and natural environment. SDOH also include structural factors associated with the societies we live in, which vary according to international location and include access to healthcare, access to and quality of food, presence of air pollution and availability of social support (120, 126). These are often aspects of life that we do not choose or have very little control over.

Many SDOH interact and intersect with each other, leading to poorer outcomes, in either an additive or even a multiplicative manner (120). High health expenditure *per capita* is more strongly associated with higher national MS prevalence than low health care expenditure *per capita* (127). This suggests that theories attributing variations in MS prevalence primarily to latitude effects on vitamin D are incomplete. However, as national wealth increases with latitude, there is likely to be an underestimation of MS prevalence in countries with low health expenditure (127). Assessing and addressing SDOH has already successfully improved outcomes in other chronic diseases and could also be beneficial in people with MS (53, 120). Unemployment has a large impact on people with MS, with worse outcomes for those who are unemployed (120). A study in France found a socioeconomic gradient in people with relapsing-onset MS, with individuals considered to live in conditions of deprivation exposed to a greater excess death rate (128).

**Figure 4:**
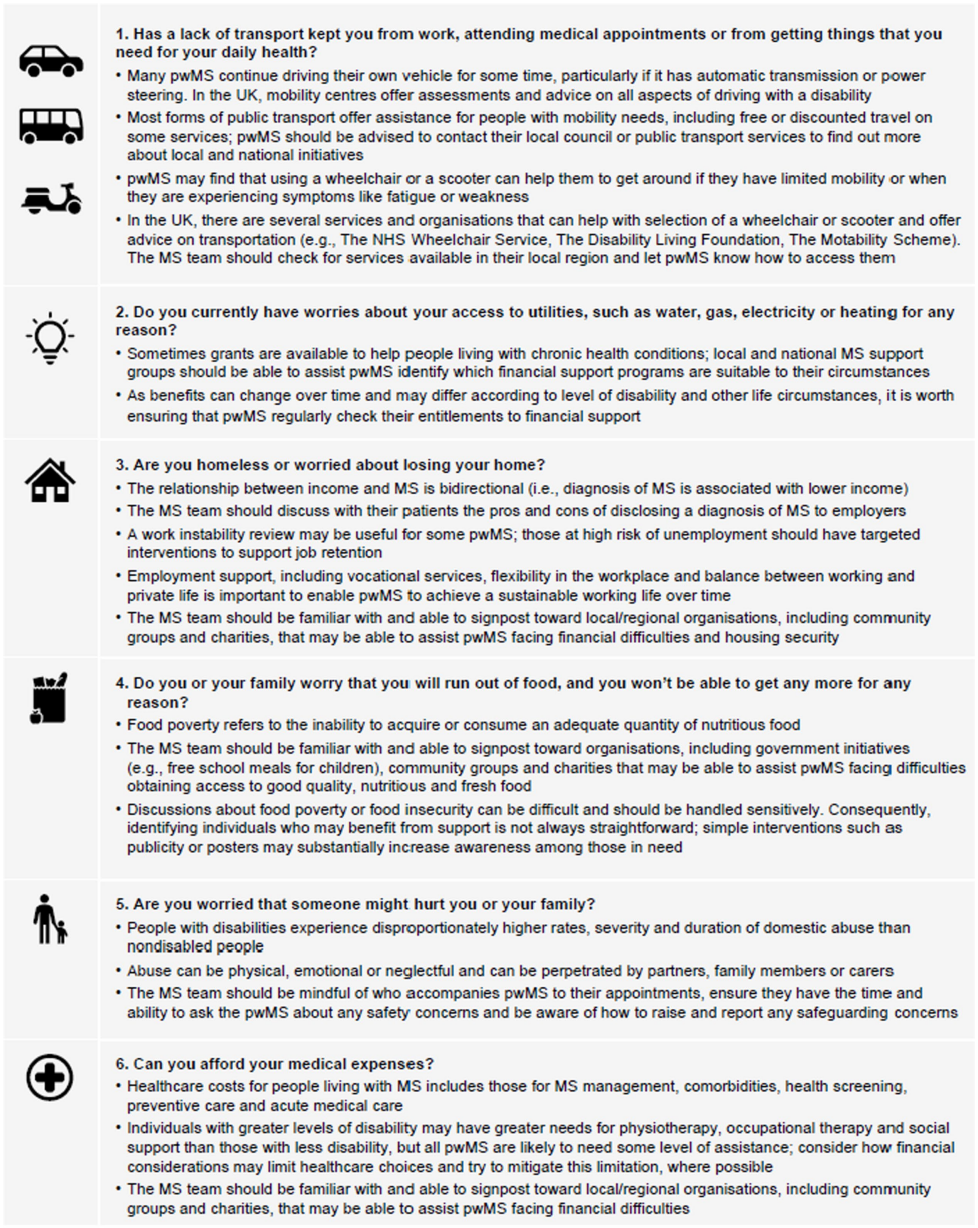
Suggested screening questions for social determinants of health in people with MS and considerations for how to offer practical support. Adapted from Bradywood et al. (130) and Dobson et al. (120). MS, multiple sclerosis; NHS, National Health Service; pwMS, people with MS; UK, United Kingdom.

Screening for SDOH is usually only performed in employment, so neurologists may not have access to this information. A validated screening tool (Core-5) exists for other health conditions (129) and an adapted version has been developed that may be useful in clinical practice for patients with MS (120). Although not all of the questions in the Core-5 tool are applicable across all healthcare and welfare systems, they provide a framework for systematic inquiry to identify individuals with the greatest need of support (120). Figure 4 presents screening questions that could be used to identify SDOH in people with MS, along with guidance on how the MS team may be able to offer support. Facilitating opportunities for people with MS to talk to each other can positively change perception of MS (131) and is a positive feature of targeted education programmes early in the disease course (Table 9).

**Table 9:** Recommendations to address social determinants of health (SDOH) in people with MS.

Regularly assess SDOH in people with MS	Try to determine SDOH for each person with MS – using the adapted Core-5 screening tool – and record the results	Screen for risk of job loss in employed people with MS	Signpost to appropriate services	Ask about SDOH at each consultation as people’s lives change
Provide people with MS with support on how to stay in employment as long as they are able or want to	Assess the main MS symptoms having an impact on work (e.g., fatigue, cognition)	Assess factors at work that are difficult for people with MS (e.g., commuting distance, access to toilets)	Refer people with MS to an employment adviser or vocational rehabilitation if they are encountering challenges at work	Support people with MS who want to stay in work for as long as possible

MS, multiple sclerosis; SDOH, social determinants of health.

## 4 Conclusion

For people with MS, early diagnosis and prompt treatment are essential components of a therapeutic strategy that offers the best chance of preserving CNS tissue and minimising, or delaying, further decline. Consequently, both an effective DMT and a “brain-healthy” lifestyle should be adopted as soon as MS is diagnosed to protect the neurological reserve and maximise lifelong brain health.

Care for people with MS should be tailored to the individual and responsive to their changing needs. To enable and maintain healthy lifestyle behaviours, people with MS may require support from family members and the broader community. It is therefore important to incorporate within routine clinical practice the ability for healthcare providers to signpost people with MS and their families to local support groups that can assist them with lifestyle behaviours (e.g., smoking cessation support, physical activity programmes, healthy eating advice). Early educational input and ongoing support for people with MS are also critical to ensure that they proactively engage in the management of their MS and have the knowledge and resources to manage comorbid conditions and adopt healthy lifestyle practices. This can help to alleviate the burden of MS and improve overall QoL.

Our recommendations, therefore, aim to preserve as much neurological reserve as possible by using an appropriate DMT to minimise disease activity early in the disease process and by adopting a “brain-healthy” lifestyle to delay disease progression. It is increasingly clear that healthy lifestyle practices should be seen as an essential form of treatment for MS, alongside pharmacologic and therapeutic medicine. A multidisciplinary, collaborative and person-centred approach to care that encompasses the healthcare team, family members and community support groups will enable people with MS to access appropriate support. In addition, there is a need for ongoing research and innovation in the field of MS to further progress and define diagnostic and therapeutic strategies, and ultimately improve outcomes.

## Author contributions

GG: Resources, Writing - review & editing. HF: Resources, Writing - review & editing. KS: Resources, Writing - review & editing. RM: Resources, Writing - review & editing. AMS: Resources, Writing - review & editing. IP: Resources, Writing - review & editing. LS: Resources, Writing - review & editing. ASc: Resources, Writing - review & editing. CB: Resources, Writing - review & editing. RS: Resources, Writing - review & editing. SH: Resources, Writing - review & editing. AW: Resources, Writing - review & editing. SJ: Resources, Writing - review & editing. CP: Resources, Writing - review & editing. ASt: Conceptualization, Resources, Writing - review & editing.
